# Investigating the triple-frequency ultrasound-assisted fermented rice lees: Impact on physicochemical, structural, morphological, and metabolic properties

**DOI:** 10.1016/j.ultsonch.2024.107176

**Published:** 2024-11-26

**Authors:** Mian Shamas Murtaza, Sanabil Yaqoob, Bismillah Mubeen, Aysha Sameen, Mian Anjum Murtaza, Abdur Rehman, Tawfiq Alsulami, Sameh A. Korma, Ibrahim Khalifa, Yong Kun Ma

**Affiliations:** a School of Food and Biological Engineering, Jiangsu University, Jiangsu, Zhenjiang, China; b Department of Food Science and Technology, MNS University of Agriculture, Multan, Pakistan; c Department of Food Science and Technology, Government College Women University, Faisalabad, Pakistan; d Institute of Food Science and Nutrition, University of Sargodha, Sargodha, Pakistan; e Department of Food Science and Nutrition, College of Food and Agricultural Sciences, King Saud University, Riyadh 11451, Saudi Arabia; f Department of Food Science, Faculty of Agriculture, Zagazig University, Zagazig 44519, Egypt; g School of Food Science and Engineering, South China University of Technology, Guangzhou 510641, China; h Food Technology Department, Faculty of Agriculture, Benha University, Moshtohor 13736, Egypt; i Department of Food Science, College of Agriculture and Veterinary Medicine, United Arab Emirates University, Al-Ain 15551, United Arab Emirates

## Abstract

This study examined the effect of triple-frequency ultrasound treatment (TFUT)-assisted lactic acid bacteria (LAB-*L. plantarum* and *L. helveticus* fermentation for 24-h and 48-h) on the chemical, structural, morphological, metabolic, and sensory properties of rice lees (RL). Ultrasonicated-assisted RL fermented with *L. helveticus* (URLH-48) had the greatest total phenolic contents (TPC) (112.1 mg GAE/m), total flavonoid contents (TFC) (163.62 mg RE/mL), and proanthocyanidin contents (PAC) (728.34 mg/mL) compared to RL (control) and other treatments. Furthermore, URLH-48 demonstrated an increase in the concentrations of quinic acid (486.96 mg/L) and gallic acid (201.42 mg/L), as determined by HPLC-UV analysis. Additionally, FTIR spectral analyses demonstrated that TFUT-assisted fermented RL exhibited a greater degree of flexibility and mobility in its secondary structures compared to RL (control). The amino acid’s profile of RL was significantly increased as LAB degraded the RL proteins, and the function of TFUT facilitates bacterial activity. Moreover, SEM observation provides convincing evidence that TFUT improves and speeds up the breakdown of proteins’ structures, resulting in irregular and dense structures. Correlation and molecular docking research suggest that TFUT has different impacts on specific RL and fermented RL characteristics. The analyses conducted using GC–MS and E-nose indicated the generation of highly volatile flavor compounds through fermentation. The sensory evaluation results show an increase in liking following fermentation and TFUT-assisted fermentation, which is attributed to the production of flavor compounds. Consequently, the combined use of TFUT-assisted fermentation markedly improves the polyphenolic composition, antioxidant capacity, flavor profile, micromorphology, and overall quality of RL, which may enhance their functionality and broaden their applications in the food industry.

## 1 Introduction

Rice lees (RL), a solid residue obtained during rice fermentation, is typically regarded as a by-product resulting from the production of certain traditional fermented foods, such as sake [1]. Sake is an alcoholic beverage that has its roots in traditional Japanese culture. In the production process, steamed rice undergoes fermentation with Aspergillus oryzae to create “koji” mold. Subsequently, koji mold, steamed rice, and Saccharomyces cerevisiae (Sake yeast) are combined, leading to the process of ethanol fermentation at the oar. Following this, the fermented product undergoes filtration, resulting in the separation of liquid and solid components. The solid component is referred to as “Sakekasu” (sake lees). Sakekasu has long been utilized in Japan as a food processing component and as a moisturizer in cosmetics because it is high in protein, peptides, amino acids, carbs, dietary fiber, fat, ash, and vitamins [2]. Sakekasu has gained popularity recently as a functional food due to its many documented benefits, including anti-colon cancer, osteoporosis prevention, and antidiabetic properties. Additionally, it reduces pain sensitivity, avoids symptoms similar to allergic rhinitis, inhibits acute alcohol-induced liver damage, and improves hepatic lipid buildup [3], [4]. Recent research has demonstrated the potential of RL as an innovative resource across several industries due to its high levels of proteins, fibers, and bioactive substances, including polyphenols and antioxidants [5].

Since antiquity, conventional fermentation methods have been investigated to improve the bioavailability of specific nutrients and advantageous metabolites, thereby augmenting the value and desirable attributes of RL [6]. The process of fermentation conducted by lactic acid bacteria (LAB) has the capacity to alter the taste and aroma of various substrates, thereby enhancing the organoleptic quality of fermented foods [7]. LAB is capable of generating significant quantities of acids, alcohols, and esters through various metabolic pathways and can produce volatile compounds originating from amino acids, peptides, and fatty acids through subsequent bioconversions [8], [9]. *L. helveticus* (NCIMB 8826), a LAB, is a Gram-positive, rod-shaped lactic acid bacterium that is frequently isolated from milk and various fermented dairy products. This bacterium is classified as an industrial homofermentative, thermophilic, non-spore-forming starter organism, and it holds a status that is generally recognized as safe [10], [11]. There is growing evidence that *L. helveticus* strains demonstrate probiotic effects and possess health-improving properties, highlighting their potential for industrial applications [12]. To address modern nutritional food demands, it is essential to develop new processing techniques that enhance the nutritional value, structural integrity, and functional properties of RL, thus enabling their effective use at the industrial level. In this context, the integration of triple-frequency ultrasound technique (TFUT) with lactic acid bacteria (LAB) fermentation may pave the way for novel avenues of exploration [13].

Ultrasound technology, especially TFUT, has garnered interest for its potential to improve the fermentation process. TFUT operates by generating acoustic cavitation, a phenomenon defined by the rapid formation and subsequent collapse of tiny bubbles, resulting in localized high temperatures and pressure gradients [14]. This leads to the disturbance of cellular architectures, improved mass transfer, and a more effective extraction of bioactive compounds [15]. The combined use of fermentation with TFUT significantly improves the release of bioactive compounds, such as phenolics and flavonoids, by facilitating the disintegration of plant cell walls and enhancing enzyme activity. The utilization of TFUT-assisted fermentation in the advancement of functional foods demonstrates a confluence of methodologies defined by in situ kindling-induced modifications, with the objective of attaining a remarkably efficient and rapid fermentation process [16]. For example, the synergistic inactivation effect and mechanism of TFUT on *A. acidoterrestris* (AAT) vegetative cells and spores, as well as on the nutrients and enzymes present in orange juice. The findings demonstrated the effective application of TFUT in fruit beverages, resulting in advantageous modifications to physical properties while maintaining the quality of ascorbic acid [17]. Similarly, in another investigation, the influence of TFUT-assisted fermentation on the phenolic content, antioxidant activity, and flavor characteristics of mulberry juice was evaluated. The antioxidant content, flavor profile, micromorphology, and overall quality of mulberry juice were enhanced by TFUT-assisted fermentation [18].

Recently, the utilization of TFUT-assisted fermentation has garnered interest as an innovative method to improve the fermentation process. Until now, TFUT-assisted RL fermentation has not been explored; however, this research delves into an innovative approach for generating high-quality, functional RL that boasts improved health advantages and greater consumer attraction. Considering the growing demand for nutrient-rich and high-quality functional food products, TFUT-assisted fermentation offers a distinctive and efficient method for producing functional RL. This approach enhances the concentration of bioactive compounds in the RL while effectively addressing challenges related to commercialization and preservation, thus fulfilling consumer demands for products that promote health and offer sensory appeal. This research aims to evaluate the influence of TFUT-assisted fermentation on the phenolic composition, antioxidant effectiveness, and sensory characteristics of RL. It can be anticipated that the combination of TFUT and fermentation will significantly increase the bioactive characteristics and consumer acceptability of RL. Moreover, our objective is to clarify the complex mechanisms that regulate the interaction between TFUT and LAB fermentation, including its impact on microstructure, flavor profile, and the bioavailability of vital metabolites.

## 2 Material and methods

### 2.1 Materials

For this study, rice lees (RL) was supplied by the fermented industry located in Zhenjiang, Jiangsu, China. After pressing and separation, the RL underwent freeze-drying to achieve a final moisture content of 3 %. The resulting freeze-dried RL were then crushed and sieved using 300 µm sieves. *Lactobacillus plantarum (L. plantarum)* and *Lactobacillus helveticus (L. helveticus),* procured from SynBio Tech, Beijing, China, were cultivated on MRS medium at a temperature of 37 °C and subsequently preserved at 4 °C. All chemicals employed in this study were of analytical grade quality.

### 2.2 Preparation of non-sonicated fermented and TFUT-treated fermented rice lees (RL)

The triple-frequency ultrasound treatment (TFUT)-assisted fermentation of RL was conducted according to the optimal conditions established in our earlier work, with slight modifications. In this experiment, *L. plantarum* and *L. helveticus*, two LAB strains that acted as inoculants, were cultivated in MRS broth in an incubator (HZQ-F160) to achieve a bacterial concentration of 107–108 CFU/mL. We used a 2 % bacterial culture for the fermentation of RL, incubating it for 48 h at 37 °C and 60 rpm. 100 g of the RL was transferred to a beaker and subjected to TFUT after 8 h of fermentation [18]. Each sample was subjected to the TFUT mode (20/28/40 kHz), employing an ultrasonic power density of 50 W/L, with a pulse length of 10 sec on and 10 sec off for a total duration of 20 min. Water recirculation was used to maintain the soaking media’s temperature at 25 ± 2 °C. Subsequent to the TFUT, the samples were placed in the incubator for 10 h of fermentation. The same procedure was repeated, using TFUT followed by fermentation after 10 h, and this process continued accordingly. The overall fermentation duration was 48 h. Finally, RL were fermented with *L. plantarum* and *L. helveticus* for 24 h and 48 h, respectively, labeled as RL (control), RLP-24, RLH-24, RLP-48, and RLH-48. Subsequently, all samples underwent TFUT and were designated as URL, URLP-24, URLH-24, URLP-48, and URLH-48.

### 2.3 Extraction of phenolics

The extraction of free phenolic compounds from non-sonicated and TFUT-treated RL fermented samples was performed according to [19]. In short, 20 mL of 80 % v/v MeOH was used to extract 1 g of the RL, and it was then sonicated for 30 min. The supernatant was lyophilized and placed as an undigested sample for further analysis following a 10-min centrifugation at 4000 g.

#### 2.3.1 Total phenolic contents (TPC)

The TPC of samples were assessed using the method described by [20] with slight modifications. In brief, a mixture was prepared by combining 1 mL of Folin-Ciocalteu (FC) reagent, 3 mL of sodium carbonate (20 % w/v), 12 mL of H_2_O, and 200 μL of the sample, followed by incubation in a water bath at 70 °C for a duration of 10 min. The absorbance was recorded at 765 nm utilizing the SpectraMax i3 spectrophotometer (Molecular Devices, Silicon Valley, CA, US). The TPC were assessed utilizing gallic acid as a standard, with the findings articulated in milligrams of gallic acid equivalent per milliliter of sample.

#### 2.3.2 Total flavonoid contents (TFC)

The TFC of the samples were measured using the methodology outlined by [20], with modifications applied. A total of 25 μl of the sample was combined with 0.1 mL of H_2_O and 10 μl of a 5 % NaNO_2_ solution, subsequently permitted to rest for a duration of 5 min. Thereafter, 15 μl of AlCl_3_ (10 %), 50 μl of NaOH, and 50 μl of H_2_O were added to the mixture. Absorbance measurements were performed at 510 nm using the SpectraMax i3 spectrophotometer (Molecular Devices, Silicon Valley, CA, US). The TFC were assessed utilizing quercetin as a reference standard, with the findings expressed in milligrams of rutin equivalent per milliliter of sample.

#### 2.3.3 Total flavonols content (TFlav)

The method described by [21] was used to calculate the TFlav of the samples, with minor adjustments. In conclusion, 2 mL of the sample, 3 mL of CH_3_COONa, and 2 mL of AlCl_3_ were combined and stored at 20 °C for 150 min. Lastly, the SpectraMax i3/spectrophotometer was used to measure the absorbance at 440 nm. Quercetin was used as a reference to calculate the TFlav, and the findings were expressed in milligrams of quercetin equivalent per milliliter of sample.

#### 2.3.4 Proanthocyanidins contents determination (PAC)

The PAC of the samples was established utilizing the approach [22], with some adjustments. After mixing 1 mL of the sample with 3 mL of HCl and 6 mL of vanillin, it was incubated for 15 min at 25 °C. The SpectraMax i3 spectrophotometer was used to measure the absorbance at 500 nm. The results were expressed in mg of catechin equivalent/mL of sample, and the PAC was calculated using catechin as a reference.

#### 2.3.5 Reducing power ability (Rp-A)

The Rp-A of the samples was ascertained through the modified methodology outlined in [23]. In summary, a mixture was prepared by combining 1 mL of the sample, 50 μl of HCl, 400 μl of C_6_N_6_FeK_3_, 400 μl of FeCl_3_, and 700 μl of H_2_O. The resultant mixture was then incubated in the dark at 37 °C for a duration of 30 min. Ultimately, the absorbance measurements were recorded at 720 nm utilizing the SpectraMax i3 spectrophotometer (SpectraMax i3). The findings were articulated in millimolar concentrations of ascorbic acid.

#### 2.3.6 Determination of ferric reducing antioxidant power (FRAP)

The evaluation of the FRAP activity in the samples was conducted employing the method outlined by [24], with certain modifications. In summary, the FRAP solution was formulated by combining 25 mL of acetate buffer (0.3 M, pH 3.6), 2.5 mL of TPTZ (10 mM, 40 mM HCl), and 2.5 mL of FeCl_3_·6H_2_O (20 mM). 0.3 mL of FRAP solution was combined with 1 mL of the sample and 0.3 mL of H_2_O. The solution was maintained at a temperature of 37 °C within a water bath for a duration of 30 min, after which the absorbance was assessed at a wavelength of 595 nm. The outcomes were determined as milligrams of trolox equivalent per milliliter of sample.

#### 2.3.7 Copper reducing power ability (Cu-Rp)

The Cu-Rp of samples was assessed using the methodology outlined by [22], with slight amendments. In brief, a mixture was prepared with 250 μl of CuCl2 (0.01 M), 250 μl of neocuproine (7.5 M), 250 μl of ammonium acetate (1 M), and varying quantities of the sample. The solution was left to stand at room temperature for 30 min, following which the absorbance was measured at 450 nm.

### 2.4 Measurement of amino acid profiling

Firstly, 2 g of the sample was mixed with 50 mL of 1 % sulfosalicylic acid. Subsequently, the extract underwent filtration using Whatman filter paper with a pore size of 0.45 μm, followed by an additional filtration through a 0.22 μm syringe membrane in preparation for chromatographic analysis. The quantification of free amino acids was conducted using the amino acid analyzer model L-8900, developed by Hitachi Co. Ltd., Tokyo, Japan. The column contained a bespoke Hitachi ion exchange resin 2622, characterized by a particle size range extending from 4.6 mm to 60 mm, with a specific particle size of 5 m. The temperature of the column fluctuated between 30 °C and 70 °C, while the reaction coil reached a notable temperature of 135 °C. Ninhydrin was employed in mobile phase A at a flow rate of 0.30 mL/min, while mobile phase B consisted of lithium citrate buffer at a flow rate of 0.35 mL/min. The calculation of amino acid content profiling was conducted utilizing the equation delineated by [25].

(1) $$X_{i}=\frac{c\times f\times V\times M}{m\times 10^{9}}\times 100$$

X_i_: Amino acid “i” content in samples (g/100 g); c: Amino acid “i” concentration in solution (nmol/mL); f: Dilution coefficient; V: Fixed specimen volume (mL); M: Amino acid “i” molar mass (g/mol); m: Sample mass (g).

### 2.5 Estimation of phenolic compounds by HPLC-UV

The HPLC-UV Agilent 1260 infinity II system was used to identify the phenolic chemicals, following a protocol similar to [19] with a few minor adjustments. Through a C-18 column (Agilent Zorbax-SB; 4.6 mm x 250 mm, 5 m particle size), the corresponding targeted analytes were eluted. Mobile phases A and B were composed of 0.1 % acetic acid and 100 % acetonitrile, respectively. 0–10 min, 5–10 % solvent B; 10–20 % solvent B; 15–25 min, 20–38 % solvent B; 25–30 min, 38–40 % solvent B; 30–31 min, 40–100 % solvent B; 31–35 min, 100 % solvent B; 35–36 min, 100–5 % solvent B; and 36–50 min, 5 % solvent B were the parameters of the linear gradient technique. The column’s temperature was maintained at 30 °C, and the flow rate was 0.8 mL min^−1^. At 250 nm, the chromatograms were captured accordingly. By comparing the retention time and spectrum of standards with samples, the phenolic compounds were identified both subjectively and quantitatively. Calibration curves were used to quantify the peak area.

### 2.6 Volatile compounds identification using HS-SPME-GC–MS

#### 2.6.1 Extraction

The extraction was performed using the Headspace Solid-Phase Microextraction technique, following the method reported in an earlier investigation [26]. 5 g of material and 1.5 g of NaCl were mixed in a 15 mL glass beaker. An internal standard of 10 μL (800 μg of 2-octanol/L) was then added into the mixture. This mixture was equilibrated at 40 °C for 20 min before the vial was sealed. The fiber composed of divinylbenzene/carboxy/polydimethylsiloxane (DVB/CAR/PDMS) with a diameter of 50/30 μm was subsequently introduced into the headspace of the glass vial, while the sample underwent continuous stirring at a frequency of 2.5 Hz throughout the duration of exposure.

#### 2.6.2 Chromatographic analysis

The study was conducted using a GC–MS (6890 N-5973B Agilent) gas chromatograph and mass spectrometer detector, equipped with a GC column measuring 60 m × 0.25 mm × 0.25 μm film thickness, constructed from Agilent J and W DBWAX (Agilent Technologies, Santa Clara, CA, USA). After extraction, the sample was positioned in the injection port. The chromatographic parameters were as follows: splitless injection mode (detection and injection temperature: 250 °C; helium as the carrier gas; flow rate of 1 mL/min); temperature profile: 10 min at 50 °C, followed by an ascent to 150 °C, then increasing at 8 °C/min until attaining 200 °C, which was sustained for 7 min. Mass spectrometry was conducted with an ion source temperature of 23 °C, a quadrupole temperature of 150 °C, and an electron impact ionization energy of 70 eV, scanning from 33 to 350 atomic mass units. The emphasis was largely on volatile chemicals [27] that exhibited over an 85 % match. Their retention indices (RI) and mass spectra were meticulously compared with those in the database of the National Institute of Standards and Technology 17 library (version 4.52, Shimadzu, Kyoto, Japan) as part of the qualitative identification process for these compounds. Chromatographic grade 2 Octanol served as the internal standard for the quantification of volatile compounds, with response and calibration factors presumed to be 1.0 [28].

### 2.7 Attenuated total reflectance-Fourier transforms infrared (ATR-FTIR) spectroscopy

The FTIR spectra for all samples were obtained using an ATR-FTIR (Thermo Scientific, Nicolet iS50, Waltham, Massachusetts, USA) thermoelectron fitted with an attenuated total reflectance (ATR) accessory that covered the range of 4000 to 700 cm^−1^ [29]. The secondary structure of all samples was identified by analyzing the observed data in the amide-I band range of 1600–1700 cm^−1^, utilizing PeakFit^TM^-V4.0 Software and OMNIC-V32 for processing.

### 2.8 Micromorphology of samples

Scanning electron micrographs of each sample were acquired at a magnification of × 2000 using the methodology outlined in [30]. Lyophilized samples were affixed to holders using double-sided tape, thereafter, coated with a gold layer, and then examined in a vacuum under a potential difference of 5 kV.

### 2.9 Molecular docking

The educational version of MOE software, 2015, USA, was used to perform the molecular docking approach. The structure of bacterial peptidoglycan (PDB ID: 2MTZ) as a macromolecule receptor and quinic acid (QA; PubChem CID: 6508) as a ligand were used to perform molecular interactions. The data were obtained from the Protein Data Bank (https://www.rcsb.org/) and PubChem Database (https://pubchem.ncbi.nlm.nih.gov/). Following the removal of H2O, structural refinement, energy reduction, and 3D protonation using MOE, both structures were optimized by merging fractional charges and minimizing energy using Protonate-3D and MMFF94X force fields. After repeating the procedure for the composites, four to five suitable docked postures were produced. These were then visualized and examined using Heatmapper (https://heatmapper.ca/expression/) for their hydrophobicity, electrostatic potential, H-bonds, and heat-map structure fluctuations. The root mean square deviation (RMSD) was then calculated after 50 ns of molecular dynamic simulation (MDs) runs, spaced 10 ns apart. With the help of the Gauss 09 program, the peptididoglycan-QA shape was initially improved using M062X operated with the 6-31G (d, p). GROMACS, version 5.1.4, GNU, Netherlands, was used to double-minimize alcalase, which was found at random surrounding peptidoglycan [31].

### 2.10 Electronic nose analysis

Electronic nose analysis of RL was carried out in accordance with the procedures described in earlier research [32]. A 50-mL sample vial containing 10 g of sample was sealed for each measurement, and an Airsense Ltd., Schwerin, Germany-based PEN3 electronic nose was employed for assessment. The following parameters were used for the measurements: internal flow rate of 400 mL/min, injection flow rate of 200 mL/min, sensor cleaning time of 150 s, sample preparation time of 5 s, and analysis duration of 120 s.

### 2.11 Sensory evaluation

The sensory evaluation was performed in the sensory laboratory of the School of Food and Biological Engineering, Jiangsu University, China. The sensory panel consists of 10 expert people (both male and female) in the age range of 20–45 years with experience of sensory evaluation. Absence of noise, uniform lighting availability, and no distracting stimuli were present in the lab. The panel was trained, and proforma was elaborated about the sensory evaluation process of the ultrasonicated-assisted fermented RL. After training, the panel established good abilities in consistently and stably conducting sensory analysis. Sensory attributes established for evaluation were as follows: (I) appearance (color), (II) aroma, (III) texture, (IV) flavor (sweet and sour), and (V) overall acceptability. These attributes were scored on a 9-point hedonic scale as (9 = like extremely; 8 = like very much; 7 = like; 6 = slightly like; 5 = neither like nor dislike; 4 = slightly dislike; 3 = dislike; 2 = very much dislike; 1 = extremely dislike). The final sensory scores of each sample were the average of the panelists’ scores.

### 2.12 Statistical analysis

The results obtained for each parameter were statistically analyzed through statistical package origin pro 8.5 and presented as mean ± standard deviations. Analysis of variance (ANOVA) and Completely Randomized Design (CRD) was performed to check the level of significance (95 %). All the analysis was performed thrice.

## 3 Results and discussion

### 3.1 Phenolics and antioxidant activity of non-sonicated and TFUT-assisted fermented RL

**Fig. 1:**
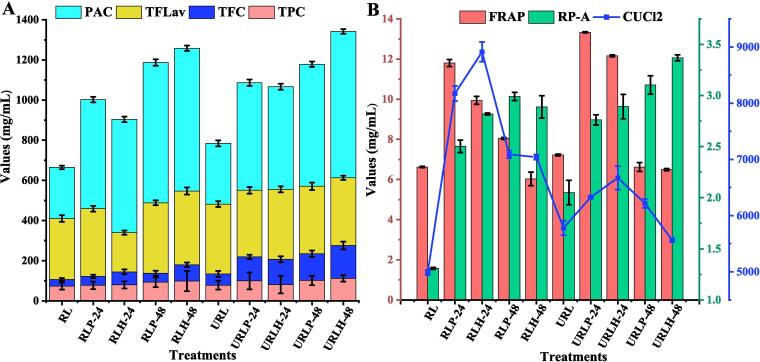
Effect of triple-frequency ultrasound assisted fermentation on the bioactive components **(A)** and their antioxidant effects **(B)**. * Rice lees (RL) were fermented with *L. plantarum* and *L. helveticus* for durations of 24 h and 48 h, resulting in the following codes: RLP-24, RLP-48, RLH-24, and RLH-48. Additionally, ultrasound-treated fermented rice lees (URL) with the same bacterial strains for 24 h and 48 h were designated as URLP-24, URLP-48, URLH-24, and URLH-48.

Fig. 1A and 1B demonstrate the results for the phenolic bioactive composition (TPC, TFC, TFlav, PAC) and antioxidant effects (CuCl2, FRAP, RP-A radical scavenging activities) of both non-sonicated and TFUT-assisted fermented RL over the 48-h fermentation period. This comprehensive analysis is essential considering the complex characteristics of phytochemical compositions and their interaction mechanisms, which cannot be sufficiently analyzed through a singular approach. Interestingly, TFUT-assisted fermentation significantly (P < 0.05) affected TPC, TFC, TFlav, and PAC in the phytochemical contents of RL, as illustrated in Fig. 1A. Ultrasonicated-assisted RL fermented with *L. helveticus* (URLH-48) presented the greatest TPC of 112.1 mg/mL, TFC of 163.62 mg/mL, and PAC of 728.34 mg/mL, whereas RL (control) displayed TPC, TFC, and PAC values of 72.92 mg/mL, 33.5 mg/mL, and 254.83 mg/mL, respectively. Surprisingly, non-sonicated RL fermented with *L. helveticus* (RLH-48) exhibited the greatest TFLav (367.5 mg/mL), followed by RL (303.33 mg/mL) and URLH-48 (337.67 mg/mL). These significant differences in TFLav align with the established variability in the depolymerization capabilities of LAB through hydrolytic enzymes and their adaptive responses [33]. The antioxidant effects of non-sonicated and TFUT-assisted fermented RL are illustrated through the reducing power ability (Rp-A), ferric reducing antioxidant power (FRAP), and copper reducing power ability (Cu-Rp) assays, as shown in in Fig. 1B. The highest FRAP value was observed in URLP-24 (13.33 mg/mL), with no significant differences identified among RL, URLP-48, and URLH-48. The highest RP-A was observed in URLH-48 at 3.369 mg/mL, in contrast to RL, which measured 1.309 mg/mL. Conversely, the concentration of CUCl_2_ increased during fermentation, reaching its peak in RLH-24 at 8912.22 mg/mL, while ultrasound-fermented samples exhibited a smaller increase. The increase in phenolic compounds is linked to the release of soluble and insoluble conjugated and bound phenolic compounds resulting from TFUT application, therefore degrading and hydrolyzing complex phytochemicals found in the RL [34]. Our findings align with [35], which also observed a comparable trend of increased phenolic content in ultrasound-fermented hawthorn pulp.

Fermentation encompasses chemical reactions that improve phenolic compounds and antioxidant activities. One process utilizes an enzyme system to release bound polyphenols, whereas the other generates organic acids, resulting in an acidic environment that reduces oxidative degradation of compounds [33], [36]. This alteration is associated with the enzymes’ degradation of complex polyphenols into simpler units during fermentation [26]. A positive correlation exists between changes in acid or estrolytic enzymes and the release of insoluble ester-bound compounds (such as phenolics and flavonoids). This phenomenon is supported by GC–MS results, which indicate that a greater quantity of esters and acids is produced in fermented samples compared to control samples [35]. Consequently, soluble conjugate phenols are released in their free form, leading to an increase in total phenol content and antioxidant activity [37]. The increased amount of antioxidant capacity in RL following fermentation and ultrasound treatment is associated with an increase in phenolic compounds, while LAB also contribute to the elevation of antioxidant capacity [38].

### 3.2 Amino acid profiling of non-sonicated and TFUT-assisted fermented RL

**Fig. 2:**
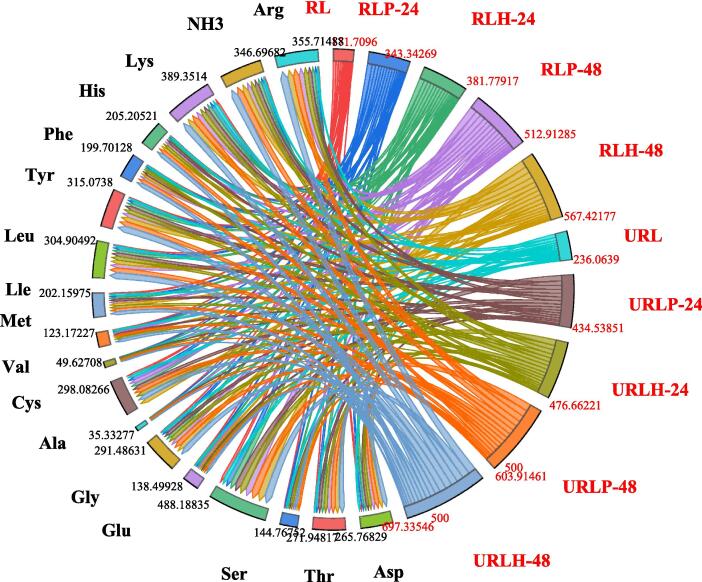
Effect of triple-frequency ultrasound assisted fermentation on the amino acid composition profiling of rice lees.

Rice protein is regarded as superior because it contains a higher quantity of essential amino acids compared to other cereals [1]. Following fermentation, starch transforms into glucose, with the primary component of the RL comprising approximately 28 % protein on a dry basis [13]. The impact of fermentation and ultrasound treatment on RL was evaluated for amino acid profiling, with the findings for seventeen free amino acids and ammonia detailed in Fig. 2. When compared to the control RL (148.41 mg/100 g), there was an increase in total free amino acids across all treatments. The ultrasonicated-assisted RL fermented with *L. helveticus* (URLH-48) yielded a maximum concentration of amino acids at 640.06 mg/100 g. The levels of both essential and non-essential amino acids have shown a significant increase because of fermentation and ultrasound treatment, with essential amino acids rising from 61.713 mg/100 g to 270.52 mg/100 g and non-essential amino acids increasing from 86.70 mg/100 g to 369.53 mg/100 g. The concentration of lysine, recognized as a limiting amino acid in cereals, increases from 6.39 mg/100 g to 34.59 mg/100 g. In the control, fermented, and ultrasound samples, higher concentrations of glutamic acid were observed (Fig. 2), which may have significantly contributed to the production of various volatile hydroxy acids, such as hexanoic acid, octanoic acid, and acetic acid, possibly influenced by aminotransferases [36], [39].

In addition to amino acids, there was a notable increase in ammonia concentration resulting from protein breakdown, rising from 20.28 ± 0.41 mg/100 g to 56.62 ± 0.40 mg/100 g. It was observed that treatments fermented with *L. helveticus* exhibit a greater increase in amino acid concentration when compared to *L. plantarum*. Proteases and peptidases are linked to *L. helveticus* and are released into the food during the process of bacterial fermentation [40]. Amino acids serve as essential components in the synthesis of proteins and play a significant role in the development of food flavors. Fermentation with *L. helveticus* results in food products that exhibit reduced bitterness and a more pronounced nutty flavor [39], [41]. Our findings are consistent with those of [42], who conducted fermentation on brown glutinous rice and noted a sustained increase in all amino acid concentrations over a period of 60 h. Ultrasounds play a significant role in the secretion and stimulation of enzymes by microbes, facilitating both intracellular and extracellular movement of substances. This process enhances the binding of substrates to enzymes, ultimately leading to improved fermentation products [36], [43].

### 3.3 Quantification of phenolic compounds in non-sonicated and TFUT-assisted fermented RL through HPLC-UV

In this experiment, ten phenolic compounds were quantified from both non-sonicated fermented and TFUT-treated fermented RL using HPLC-UV, with the results presented in Table 1. The data was examined by quantifying the peak area at a designated retention time relative to the standards in the spectra of various samples. Table 1 demonstrates that both fermentation and TFUT exhibit a favorable and statistically significant effect (p < 0.05) on the phenolic compounds of RL, with all components exhibiting an increase throughout both processes. The total phenolic compounds in the RL (control sample) measured 152.25, but after 48 h of TFUT-assisted fermentation with *L. helvaticus* (URLH-48), their total concentration was increased to 791.28. The TFUT-treated fermented samples demonstrated a more significant increase in phenolic compounds than the non-sonicated fermented samples. The most significant rise was observed in gallic and quinic acids, respectively. Vanilic acid was identified as the least prevalent among all the phenolic compounds. These results are aligned with the measurement of phenolic and flavonoid compounds (Fig. 1).

**Table 1:** Phenolic acids of fermented and ultrasound assisted fermented Rice wine lees through HPLC-UV.

**Compound**	**Chemical Formula**	**RT (min)**	**RL**	**RLP-24**	**RLH-24**	**RLP-48**	**RLH-48**	**URL**	**URLP-24**	**URLH-24**	**URLP-48**	**URLH-48**
Cafeic Acid	C_9_H_10_O_5_	17.22	0.72 ± 0.03^g^	1.46 ± 0.11^f^	2.18 ± 0.08^e^	3.30 ± 0.17^d^	4.00 ± 0.22^b^	0.93 ± 0.19^g^	3.79 ± 0.05^c^	3.23 ± 0.11^d^	4.28 ± 0.23^a^	4.16 ± 0.18^ab^
Coumeric Acid	C_9_H_8_O_3_	20.3	1.87 ± 1.61^i^	2.09 ± 1.12^h^	2.70 ± 0.32^g^	3.04 ± 0.14^f^	5.14 ± 0.09^e^	2.70 ± 0.22^g^	13.50 ± 0.15^d^	16.04 ± 0.07^c^	18.68 ± 0.23^b^	19.73 ± 0.14^a^
Gallic Acid	C_7_H_6_OH_5_	3.24	31.55 ± 2.23^g^	119.01 ± 2.75^e^	130.21 ± 1.67^d^	132.04 ± 0.99^d^	127.35 ± 1.25^de^	40.47 ± 1.42^f^	190.88 ± 0.83^c^	210.37 ± 0.54^a^	201.23 ± 0.62^b^	201.42 ± 1.48^b^
Chlorogenic acid	C_16_H_18_O_9_	14.90	0.55 ± 0.06^f^	2.99 ± 0.11^e^	2.81 ± 0.17^e^	6.65 ± 0.21^ab^	6.15 ± 0.34^b^	0.73 ± 0.27^f^	4.54 ± 0.09^d^	4.77 ± 0.15^d^	5.06 ± 0.20^c^	6.91 ± 0.11^a^
Quinic Acid	C_7_H_12_O_6_	33.95	109.12 ± 2.34^h^	143.74 ± 3.06^g^	203.61 ± 2.01^f^	217.17 ± 1.78^ef^	307.68 ± 2.95^d^	233.61 ± 3.5^e^	318.19 ± 2.83^d^	372.04 ± 3.72^c^	396.91 ± 1.76^b^	486.96 ± 1.83^a^
Ferulic acid	C_10_H_10_O_4_	21.40	1.25 ± 0.05^h^	1.58 ± 0.12^fg^	1.42 ± 0.11^g^	3.15 ± 0.20^d^	2.38 ± 0.14^e^	1.84 ± 0.06^f^	5.03 ± 0.10^c^	5.12 ± 0.09^c^	8.63 ± 0.22^a^	8.05 ± 0.17^b^
Procatenoic Acid	C_7_H_6_O_4_	28.30	1.06 ± 0.17^g^	1.90 ± 0.06^f^	2.07 ± 0.14^d^	1.99 ± 0.14^de^	2.01 ± 0.21^de^	1.95 ± 0.18^e^	5.36 ± 0.09^b^	5.20 ± 0.13^b^	3.41 ± 0.16^c^	6.08 ± 0.11^a^
Vanilic acid	C_8_H_8_O_4_	16.15	0.75 ± 0.04^g^	1.69 ± 0.12^f^	1.86 ± 0.09^f^	2.46 ± 0.23^d^	2.36 ± 0.21^de^	0.99 ± 0.30^g^	2.27 ± 0.18^e^	2.74 ± 0.22^c^	3.68 ± 0.29^b^	4.62 ± 0.15^a^
Neochlorogenic acid	C_16_H_18_O_9_	10.11	2.55 ± 0.13^g^	27.09 ± 0.36^de^	26.23 ± 0.27^e^	39.36 ± 0.19^b^	32.05 ± 0.41^c^	9.53 ± 0.16^f^	27.85 ± 0.33^d^	33.36 ± 0.32^c^	39.10 ± 0.24^b^	42.64 ± 0.19^a^
Trihydroxy Benzoic Acid	C_6_H_2_ (OH)_3_CO_2_H	8.22	2.84 ± 0.20^h^	4.31 ± 0.27^g^	5.36 ± 0.31^f^	6.48 ± 0.18^de^	6.95 ± 0.22^d^	5.95 ± 0.32^ef^	10.39 ± 0.16^c^	10.29 ± 0.19^c^	15.12 ± 0.23^a^	10.72 ± 0.31^b^
Total			152.25 ± 1.15^h^	305.86 ± 2.11^g^	378.46 ± 2.46^fg^	415.66 ± 1.06^f^	496.07 ± 2.32^e^	298.69 ± 1.7^g^	581.81 ± 0.99^d^	663.15 ± 1.73^c^	696.11 ± 1.85^b^	791.28 ± 2.24^a^

* Rice lees (RL) were fermented with L. plantarum and L. helveticus for durations of 24 h and 48 h, resulting in the following codes: RLP-24, RLP-48, RLH-24, and RLH-48. Additionally, ultrasound-treated fermented rice lees (URL) with the same bacterial strains for 24 h and 48 h were designated as URLP-24, URLP-48, URLH-24, and URLH-48.

Different enzymatic reactions occur during fermentation, leading to an increase in phenolic compounds. The fermentation process activates hydrolytic enzymes, which ultimately lead to the release of phenolic compounds from the matrix [44]. For example, glucose acts as a fundamental component for the commencement of phenolic synthesis. It undergoes glycolysis and the phenyl propane pathway, which are responsible for converting and creating new phenolic compounds [45]. The enzyme *β*-glucosidase is responsible for breaking down glycosidic bonds, which aids in the release of phenolic glycosides [46]. Certain phenolic compounds, such as caffeic and ferulic acids, exhibit ester bonding both before and during the fermentation process. Ultrasonic treatment has the ability to release these phenolic acids from their chemically bound state [47]. As mentioned previously, phytochemicals are associated with polysaccharides and are released gradually, while multifrequency ultrasound treatment enhances the breaking of chemical bonds and the dissociation of phenolic acid into their derivatives, resulting in the release of phenolic components; thus, TFUT-assisted fermentation facilitates a greater release of bound polyphenols [48]. The results of our study align with the findings of [49], which examine the impact of LAB fermentation on various milling fractions of rice bran.

### 3.4 Volatile compounds profiling of non-sonicated and TFUT-assisted fermented RL using HS-SPME/GC–MS

The impact of fermentation and ultrasound-assisted fermentation was evaluated through analytical methods, specifically HS-SPME coupled with GC–MS, with the findings detailed in Table 2. A total of 48 aromatic compounds were identified across various treatments, with the following quantities observed: 23 in RL, 28 in RLP-24, 31 in RLH-24, 33 in RLP-48, 32 in RLH-48, 28 in URL, 26 in URLP-24, 29 in URLH-24, 26 in URLP-48, and 32 in URLH-48. The identified compounds exhibited concentrations of 1205.16, 1304.45, 1443.14, 1482.72, 1638.01, 1496.22, 1342.87, 1416.42, 1507.67, and 1698.93 across treatments RL, RLP-24, RLH-24, RLP-48, RLH-48, URL, URLP-24, URLH-24, URLP-48, and URLH-48, respectively. The highest levels of esters, alcohol, and acids were identified in RLH-48 (800.20 ng/100 g), URLH-48 (827.53 ng/100 g), and RLH-48 (91.77 ng/100 g), respectively. Additionally, the overall peak concentration of compounds was observed in URLH-48 (1698.93 ng/100 g). Esters were identified as a prominent class, exhibiting concentrations of 47.30 %, 48.38 %, 50.72 %, 48.51 %, 48.84 %, 49.79 %, 48.43 %, 48.58 %, 44.79 %, and 44.81 % across various treatments, with alcohols and acids generated during fermentation following in prevalence. The results demonstrated a notable alignment with earlier studies conducted by [33] and [50]. The GC–MS analysis results correspond with the qualitative and quantitative outcomes of the E-nose system and the radar development, demonstrating that *L. halvaticus* generates the highest levels of volatile flavoring compounds after 48 h in both fermented and ultrasound-treated samples.

**Table 2:** Quantitative amounts of volatile compounds identified in fermented, and ultrasound assisted fermented rice wine lees by HS-SPME-GC/MS method.

**Name of Compound**	**Retention Time (min)**	**CAS #**	**RL**	**RLP-24**	**RLH-24**	**RLP-48**	**RLH-48**	**URL**	**URLP-24**	**URLH-24**	**URLP-48**	**URLH-48**
**Acids**												
Hexanoic acid	23.33	142–62-1	3.17 ± 0.11^d^	6.96 ± 0.23^bc^	7.48 ± 0.52^b^	20.59 ± 0.21^a^	20.62 ± 0.36^a^	5.69 ± 0.05^c^	n. d	8.92 ± 0.34^a^	8.77 ± 0.11^ab^	8.50 ± 0.32^ab^
Acetic acid	13.96	64–19-7	n. d	42.22 ± 0.09^e^	44.68 ± 0.68^e^	56.73 ± 0.38^b^	57.21 ± 0.19^b^	n. d	51.03 ± 0.20^c^	52.55 ± 0.21^c^	50.21 ± 0.23^d^	59.82 ± 0.45^a^
Propanoic acid, 2-methyl	16.99	79–31-2	n. d	n. d	8.43 ± 0.23^a^	5.56 ± 0.05^c^	6.58 ± 0.31^b^	4.42 ± 0.03^d^	n. d	n. d	n. d	n. d
Hexadecanoic acid	41.96	57–10-3	n. d	n. d	n. d	8.11 ± 0.27^a^	7.36 ± 0.13^b^	n. d	n. d	n. d	n. d	n. d
Oxime, methoxy phenyl	21.97	0–0–0	n. d	n. d	n. d	n. d	n. d	1.07 ± 0.17	13.54 ± 0.16	16.46 ± 0.57	11.33 ± 0.06	10.03 ± 0.33
**Sub Total**			3.17 ± 0.11^g^	49.18 ± 0.16^e^	60.59 ± 0.42^d^	90.99 ± 0.74^a^	91.77 ± 0.32^a^	11.17 ± 0.06^f^	64.57 ± 0.18^d^	77.93 ± 0.41^b^	70.31 ± 0.10^c^	78.35 ± 0.37^b^
**Alcohols**												
1-Butanol, 3-methyl	7.64	123–51-3	216.91 ± 0.41^f^	217.66 ± 0.37^f^	214.85 ± 0.54^g^	225.54 ± 0.06^d^	227.81 ± 0.21^d^	253.99 ± 0.18^c^	224.31 ± 0.33^d^	222.15 ± 0.41^e^	276.92 ± 0.42^b^	305.78 ± 0.26^a^
1-Butanol, 3-methyl-, acetate	5.27	123–92-2	15.21 ± 0.13^a^	10.23 ± 0.19^b^	11.99 ± 0.03^b^	8.77 ± 0.25^c^	9.31 ± 0.31^bc^	16.51 ± 0.27^a^	9.22 ± 0.23^bc^	8.16 ± 0.34^c^	4.11 ± 0.45^e^	5.11 ± 0.31^d^
1-Hexanol	11.60	111–27-3	3.96 ± 0.24^d^	5.42 ± 0.13^bc^	5.38 ± 0.18^bc^	5.72 ± 0.07^b^	5.78 ± 0.17^b^	4.67 ± 0.22^c^	3.99 ± 0.26^d^	4.52 ± 0.21^c^	6.86 ± 0.16^a^	7.01 ± 0.14^a^
1-Propanol, 2-methyl-	4.76	78–83-1	51.74 ± 0.46^a^	22.11 ± 0.31^g^	25.48 ± 0.22^f^	27.56 ± 0.25^e^	29.36 ± 0.31^d^	35.40 ± 0.35^b^	27.43 ± 0.42^e^	28.78 ± 0.31^d^	32.71 ± 0.28^c^	33.72 ± 0.08^c^
2,3-Butanediol, [R- (R*, R*)]	16.38	24347–58-8	45.88 ± 0.73^e^	19.73 ± 0.35^h^	23.90 ± 0.55^g^	64.22 ± 0.21^bc^	67.72 ± 0.04^b^	61.26 ± 0.16^c^	35.22 ± 0.18^f^	38.83 ± 0.25^f^	55.45 ± 0.21^d^	71.70 ± 0.34^a^
Phenylethyl Alcohol	24.66	60–12-8	278.50 ± 0.21^f^	311.11 ± 0.04^d^	337.08 ± 0.09^c^	303.34 ± 0.19^de^	340.81 ± 0.42^bc^	332.27 ± 0.35^c^	288.34 ± 0.27^e^	291.59 ± 0.21^e^	344.98 ± 0.26^b^	391.22 ± 0.22^a^
2,3-Butanediol	17.31	513–85-9	n. d	n. d	n. d	n. d	34.89 ± 0.73^a^	n. d	n. d	n. d	n. d	n. d
2-Octenal	13.27	2548–87-0	n. d	6.88 ± 0.22^a^	2.09 ± 0.07^b^	1.67 ± 0.53^b^	n. d	n. d	n. d	n. d	n. d	n. d
1-Octanol	16.92	111–87-5	n. d	n. d	n. d	n. d	n. d	n. d	n. d	6.38 ± 0.08^a^	n. d	n. d
Pentane, 1-Nitro	5.20	628–5-7	n. d	n. d	n. d	n. d	n. d	n. d	n. d	n. d	n. d	5.05 ± 0.26^a^
1-Propanol, 3-(methylthio)	20.46	505–10-2	n. d	n. d	n. d	n. d	n. d	n. d	n. d	n. d	n. d	4.06 ± 0.12^a^
2-Octanol	13.48	123–96-6	n. d	n. d	n. d	n. d	n. d	n. d	n. d	n. d	n. d	3.88 ± 0.31^a^
**Sub Total**			612.20 ± 0.40^e^	592.52 ± 0.32^f^	620.77 ± 0.57^de^	636.82 ± 0.18^d^	715.68 ± 0.66^bc^	704.10 ± 0.27^c^	588.51 ± 0.91^f^	600.40 ± 0.38^e^	721.03 ± 0.51^b^	827.53 ± 0.62^a^
**Esters**												
(E)-9-Octadecenoic acid ethyl ester	37.39	6114–18-7	29.80 ± 0.23^e^	31.33 ± 0.51^de^	35.78 ± 0.07^b^	32.56 ± 0.13^d^	36.86 ± 0.28^a^	35.33 ± 0.42^b^	34.64 ± 0.11^c^	35.69 ± 0.17^b^	29.66 ± 0.46^e^	32.98 ± 0.33^d^
Acetic acid, 2-phenylethyl ester	22.65	103–45-7	15.59 ± 0.14^e^	18.63 ± 0.21^c^	20.71 ± 0.09^b^	17.21 ± 0.16^cd^	20.68 ± 0.26^b^	25.35 ± 0.21^a^	14.44 ± 0.33^f^	16.93 ± 0.28^d^	17.23 ± 0.42^cd^	16.62 ± 0.51^d^
Butanedioic acid, diethyl ester	19.71	123–25-1	14.89 ± 0.61^ef^	14.55 ± 0.24^ef^	18.68 ± 0.31^d^	15.43 ± 0.31^e^	17.18 ± 0.18^de^	20.63 ± 0.26^c^	8.66 ± 0.11^g^	10.71 ± 0.37^f^	22.22 ± 0.21^b^	29.78 ± 0.43^a^
Decanoic acid, ethyl ester	18.99	110–38-3	114.98 ± 0.33^g^	132.45 ± 0.16^f^	145.68 ± 0.20^de^	151.33 ± 0.52^c^	171.21 ± 0.08^a^	146.37 ± 0.27^de^	139.33 ± 0.29^e^	149.22 ± 0.16^d^	148.78 ± 0.27^d^	165.09 ± 0.39^b^
Dodecanoic acid, ethyl ester	23.62	106–33-2	14.62 ± 0.22^f^	22.24 ± 0.17^e^	29.28 ± 0.09^d^	37.66 ± 0.23^a^	35.91 ± 0.30^b^	31.33 ± 0.21^c^	28.25 ± 0.25^d^	30.95 ± 0.23^c^	35.53 ± 0.15^b^	37.62 ± 0.17^a^
Ethyl 9-hexadecenoate	34.54	54546–22-4	6.16 ± 0.14^d^	7.44 ± 0.18^c^	9.64 ± 0.07^a^	7.01 ± 0.22^c^	6.55 ± 0.15^cd^	6.07 ± 0.20^d^	4.67 ± 0.19^e^	3.29 ± 0.08^f^	5.88 ± 0.11^d^	8.40 ± 0.23^b^
Hexadecanoic acid, ethyl ester	34.21	628–97-7	203.23 ± 0.61^e^	210.87 ± 0.29^d^	234.60 ± 0.35^b^	218.66 ± 0.17^c^	231.31 ± 0.57^bc^	256.84 ± 0.44^a^	222.56 ± 0.25^c^	231.57 ± 0.18^bc^	224.79 ± 0.26^c^	236.07 ± 0.32^b^
Hexanoic acid, ethyl ester	8.28	123–66-0	11.51 ± 0.42^a^	n. d	n. d	n. d	n. d	n. d	n. d	n. d	n. d	9.67 ± 0.33^b^
Linoleic acid ethyl ester	39.03	544–35-4	25.53 ± 0.26^g^	28.96 ± 0.17^f^	34.01 ± 0.72^c^	32.32 ± 0.51^d^	40.54 ± 0.44^b^	46.87 ± 0.41^a^	30.18 ± 0.18^e^	33.98 ± 0.26^c^	34.93 ± 0.24^c^	44.76 ± 0.31^ab^
Methoxyacetic acid, hexyl ester	9.43	145747–16-6	1.64 ± 0.12^a^	n. d	n. d	n. d	n. d	n. d	n. d	n. d	n. d	n. d
Octanoic acid, ethyl ester	13.87	106–32-1	92.33 ± 0.14^f^	109.12 ± 0.29^d^	122.63 ± 0.20^b^	125.83 ± 0.22^b^	157.66 ± 0.21^a^	114.40 ± 0.07^c^	110.29 ± 0.17^d^	112.27 ± 0.18^cd^	99.93 ± 0.23^e^	101.59 ± 0.31^e^
Tetradecanoic acid, ethyl ester	29.37	124–6-1	39.77 ± 0.30^e^	51.30 ± 0.26^d^	58.10 ± 0.13^c^	64.22 ± 0.06^a^	65.27 ± 0.27^a^	58.17 ± 0.31^c^	55.50 ± 0.21^cd^	59.94 ± 0.27^c^	52.39 ± 0.31^d^	62.80 ± 0.15^b^
Dimethyl phthalate	34.33	131–11-3	n. d	n. d	15.19 ± 0.14^a^	6.77 ± 0.21^c^	2.23 ± 0.26^d^	n. d	n. d	n. d	n. d	12.36 ± 0.09^b^
Heptanoic acid, ethyl ester	10.88	106–30-9	n. d	0.96 ± 0.07^c^	1.72 ± 0.13^b^	1.12 ± 0.11^bc^	2.31 ± 0.20^a^	n. d	n. d	n. d	n. d	n. d
Octadecanoic acid, ethyl ester	37.19	111–61-5	n. d	3.45 ± 0.13^a^	2.74 ± 0.18^b^	1.97 ± 0.20^e^	2.10 ± 0.09^d^	2.37 ± 0.11^c^	2.01 ± 0.15^de^	2.26 ± 0.16^c^	1.93 ± 0.22^e^	2.36 ± 0.23^c^
Propanoic acid, 2-hydroxy-, ethyl ester	11.32	97–64-3	n. d	n. d	2.11 ± 0.17^cd^	4.44 ± 0.23^b^	6.13 ± 0.12^a^	n. d	n. d	n. d	2.25 ± 0.33^c^	1.81 ± 0.28^d^
Pentyl octanoate	19.45	638–25-5	n. d	n. d	2.02 ± 0.18^a^	n. d	n. d	n. d	n. d	n. d	n. d	n. d
Ethyl 9-decenoate	20.05	67233–91-4	n. d	n. d	n. d	3.00 ± 0.23^b^	4.27 ± 0.16^a^	n. d	n. d	n. d	n. d	n. d
Nonanoic acid, ethyl ester	16.28	123–29-5	n. d	n. d	n. d	n. d	n. d	2.09 ± 0.11^a^	n. d	1.86 ± 0.06^b^	n. d	n. d
**Sub Total**			570.03 ± 0.32^h^	631.30 ± 0.28^g^	732.88 ± 0.40^c^	719.53 ± 0.29^d^	800.20 ± 0.53^a^	745.82 ± 0.44^b^	650.53 ± 0.26^f^	688.67 ± 0.37^e^	675.52 ± 0.24^ef^	761.88 ± 0.19^b^
**Ketones**												
Acetoin	9.70	513–86-0	n. d	8.22 ± 0.31^b^	11.65 ± 0.62^a^	6.87 ± 0.21^c^	8.13 ± 0.14^b^	2.28 ± 0.05^d^	1.89 ± 0.24^d^	2.17 ± 0.76^d^	n. d	n. d
4-Hydroxy-2-butanone	20.99	590–90-9	n. d	2.58 ± 0.25^a^	2.69 ± 0.35^a^	n. d	n. d	n. d	1.17 ± 0.63^b^	n. d	2.05 ± 0.17^ab^	1.99 ± 0.32^ab^
**Sub Total**			0.00	10.80 ± 0.27^b^	14.34 ± 0.44^a^	6.87 ± 0.21^d^	8.13 ± 0.14^c^	2.28 ± 0.05^f^	3.06 ± 0.45^e^	2.17 ± 0.76^f^	2.05 ± 0.17^f^	1.99 ± 0.32^f^
**Aldehydes**												
Benzaldehyde	15.66	100–52-7	4.75 ± 0.61^d^	3.89 ± 0.36^e^	3.77 ± 0.08^ef^	3.43 ± 0.14^f^	3.22 ± 0.32^f^	5.63 ± 0.22^d^	11.12 ± 0.27^b^	13.60 ± 0.41^a^	7.67 ± 0.49^c^	8.34 ± 0.34^c^
Benzeneacetaldehyde	18.58	122–78-1	5.37 ± 0.44^b^	n. d	n. d	n. d	n. d	5.73 ± 0.27^a^	n. d	n. d	n. d	n. d
Hexanal	4.31	66–25-1	n. d	2.99 ± 0.26^d^	3.19 ± 0.19^cd^	4.16 ± 0.08^b^	5.14 ± 0.17^a^	1.93 ± 0.24^e^	3.56 ± 0.31^c^	3.33 ± 0.20^cd^	4.04 ± 0.21^b^	3.74 ± 0.15^bc^
Nonanal	12.31	124–19-6	n. d	n. d	n. d	n. d	n. d	n. d	n. d	2.52 ± 0.16^a^	n. d	n. d
**Sub Total**			10.13 ± 0.49^d^	6.88 ± 0.29^f^	6.96 ± 0.12^f^	7.59 ± 0.11^ef^	8.36 ± 0.26^e^	13.29 ± 0.25^b^	14.68 ± 0.29^b^	19.45 ± 0.26^a^	11.71 ± 0.30^c^	12.08 ± 0.27^c^
**Others**												
Benzofuran, 2,3-dihydro	35.79	496–16-2	3.11 ± 0.14^c^	3.98 ± 0.20^ab^	4.42 ± 0.08^a^	3.44 ± 0.31^bc^	3.92 ± 0.22^ab^	3.89 ± 0.25^ab^	2.09 ± 0.31^d^	3.48 ± 0.27^bc^	3.18 ± 0.09^c^	3.78 ± 0.11^b^
Caryophyllene	17.66	87–44-5	6.51 ± 0.31^a^	5.33 ± 0.25^ab^	n. d	n. d	n. d	n. d	n. d	n. d	n. d	4.39 ± 0.17^b^
2-Bromo dodecane	18.27	13187–99-0	n. d	4.46 ± 0.61^a^	3.19 ± 0.35^b^	n. d	n. d	n. d	n. d	n. d	n. d	n. d
Silanediol, dimethyl	19.19	1066–42-8	n. d	n. d	n. d	8.54 ± 0.37^b^	9.94 ± 0.29^a^	3.07 ± 0.07^c^	2.77 ± 0.18^cd^	2.42 ± 0.26^d^	n. d	n. d
Ammonium acetate	14.04	631–61-8	n. d	n. d	n. d	8.94 ± 0.24^e^	n. d	12.59 ± 0.09^d^	16.66 ± 0.16^c^	21.90 ± 0.42^b^	23.87 ± 0.27^a^	n. d
1-Fluorooctane	16.98	463–11-6	n. d	n. d	n. d	n. d	n. d	n. d	n. d	n. d	n. d	8.93 ± 0.16^a^
**Sub Total**			9.63 ± 0.22^f^	13.77 ± 0.31^e^	7.61 ± 0.18^g^	20.92 ± 0.27^bc^	13.86 ± 0.25^e^	19.55 ± 0.14^c^	21.52 ± 0.21^b^	27.80 ± 0.30^a^	27.05 ± 0.15^a^	17.10 ± 0.14^d^
**Grand Total (48)**			1205.16 ± 0.53^i^	1304.45 ± 0.73^h^	1443.14 ± 0.68^e^	1482.72 ± 0.26^d^	1638.01 ± 0.71^b^	1496.22 ± 0.27^cd^	1342.87 ± 0.41^g^	1416.42 ± 0.29^f^	1507.67 ± 0.33^c^	1698.93 ± 0.42^a^

Among esters, the ethyl ester stands out as the most prevalent, exhibiting a peak concentration of 236.07 ng/100 g. Seven novel esters have been identified: dimethyl phthalate, heptanoic acid ethyl ester, octadecanoic acid ethyl ester, propanoic acid 2-hydroxy ethyl ester, and pentyl octanoate. During fermentation, ethyl 9-decenoate, nonanoic acid, and ethyl ester emerged, which were not present in the RL (control sample). These compounds may serve as the foundation for the evolution of flavor, contributing fruity, woody, and spicy notes in the samples [51]. A significant difference was noted in the esters; the concentration of esters in the control group was 570.03 ng/100 g, which rose to 800.20 ng/100 g in RLH-48 (Table 2). The notable rise is responsible for the fruity aroma observed in the fermented samples. The results indicate a significant enhancement in aroma compounds when compared with earlier studies focused on the fruit during its ripening phases [52], [53].

Esterification reactions involve the transformation of alcohols and acids to yield esters. Alternatively, the enzyme alcohol acetyltransferase facilitates the production of increased amounts of acetyl-CoA and alcohol. Notably, even minor fluctuations in the concentration of secondary metabolites can profoundly influence sensory parameters [54]. Initially, the RL contained a singular acid; however, following the fermentation process, an additional four acids emerged, namely acetic acid, propanoic acid, 2-methyl, hexadecanoic acid, oxime, and methoxyphenyl. Furthermore, the concentration of hexanoic acid exhibited a notable increase, rising from 3.17 ng/100 g in the control to 20.62 ng/100 g in RLH-48. An extraordinary elevation in acid concentration was observed, rising from 3.17 ng/100 g to 91.77 ng/100 g. Following fermentation and ultrasonic treatment, six new alcohols were detected in the samples: 2,3-Butanediol, 2-Octenal, 1-Octanol, Pentane, 1-Nitro, 1-Propanol, 3-(methylthiol), and 2-Octanol (Table 2). The highest observed increase in alcohol was recorded at 827.53 ng/100 g in URLH-48. Alcohols play a crucial role in the fermentation process and can be produced through various biochemical pathways, including glycolysis, amino acid metabolism, the degradation of linoleic and linolenic acids, as well as the reduction of methyl ketones [54].

The production of aldehydes resulted in two additional aldehydes not found in the RL (control sample), while fermentation resulted in an increase in the concentration of existing aldehydes, including benzaldehyde. The increase in benzeneacetaldehyde and benzaldehyde may play a significant role in enhancing fruity sensory characteristics, including flavors reminiscent of cherry, almond, or vanilla [51]. The bioconversion of aldehydes was observed to be linked to the auto-oxidation of unsaturated fatty acids, as identified by [53]. The control sample exhibited a significant absence of two ketones, acetoin and 4-hydroxy-2-butanone, which were produced via fermentation and ultrasound treatment. URLH-48 is the sole sample that contains fluorooctane, which contributes to the flower-like aroma and likely serves as the main factor for its highest scores in the sensory evaluation [55]. The results indicate that ultrasound treatment significantly influenced the volatile compounds, and meticulously treated samples can enhance the quantity of volatile compounds. Ultrasounds have the potential to liberate additional fragrant substances that are encapsulated within the cells and may also enhance molecular structural modifications [35]. The findings align closely with those of [54], who investigated the impact of ultrasound-assisted fermentation and observed a comparable pattern of volatile compounds.

### 3.5 FTIR spectra of non-sonicated fermented and TFUT-assisted fermented RL

**Fig. 3:**
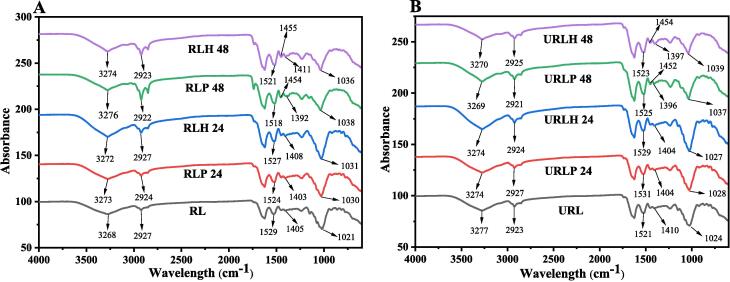
FTIR peaks of non-sonicated fermented samples at 24 and 48 h (A) and TFUT-assisted fermented RL at 24 and 48 h (B).

FTIR spectroscopy illustrates the vibrational spectrum of molecules, offering an accurate representation of their secondary structure. Metabolic fingerprints serve as a visual representation of spectral data, offering a comprehensive understanding of the environment and molecular structure via specific vibrational modes [14]. The impact of fermentation and TFUT-assisted fermentation on the RL secondary structure was evaluated using FTIR, with the resulting data displayed as peaks in Fig. 3, alongside the control sample for comparison. The non-sonicated fermented and TFUT-assisted fermented samples exhibited a lower intensity of the –OH peak (3268 cm^−1^) compared to the control (RL) samples. This observation is associated with alterations in hydrogen bonding. During fermentation, components undergo breakdown and structural changes in their functional groups, while TFUT weakens the interactions among protein chains. The disruption of hydrogen bonds results in the release of hydroxyl ions, subsequently enhancing the intensity of the absorption peak [56]. The absence of an absorbance peak in the spectral range of 2000 to 2500 cm^−1^ indicates the non-existence of chemical groups such as C≡N, C≡C, or C≡C≡C clusters. Observations were made regarding the changes in the absorption peaks associated with N-O stretching, S = O stretching, and C-N stretching vibrations at 1532 cm^−1^ (amide I), 1405 cm^−1^ (amide II), and 1021 cm^−1^ (amide III), respectively [57].

The intensity of the absorption peaks for amides I, II, and III is enhanced following ultrasound treatment, and fermentation is observed to have a beneficial effect. Ultrasound application alters the structure and distribution of functional groups within the protein matrix while not affecting the type of functional groups present [50]. Fermentation negatively impacts polysaccharides such as glucose, cellulose, and hemicellulose within the range of 500–1500 cm^−1^ [58]. RL (control sample) exhibits fewer distinct peaks, as illustrated in Fig. 3, indicating reduced C-O-C ether stretching within the 500–1000 cm^−1^ wavenumber range [59]. Following 48 h of fermentation, a C-H bending alkane group was observed between 1452 cm^−1^ and 1455 cm^−1^ in only four TFUT-assisted fermented samples (URLP-24, URLP-48, URLH-24, and URLH-48).

The consequences of fermentation lead to the breakdown of various compounds and the production of new ones. This outcome is associated with the results of GC, which indicate the formation of numerous acids, esters, aldehydes, and ketones. Our results correlate with the findings of [60], which examine the impact of thermal and ultrasound treatment on soy protein isolates, revealing a shift in peaks attributed to the influence of TFUT. The results of an earlier study [59] also align with our findings, which utilized different LAB and yeast for fermentation and concluded that there is a shifting of functional groups throughout the fermentation period.

**Fig. 4:**
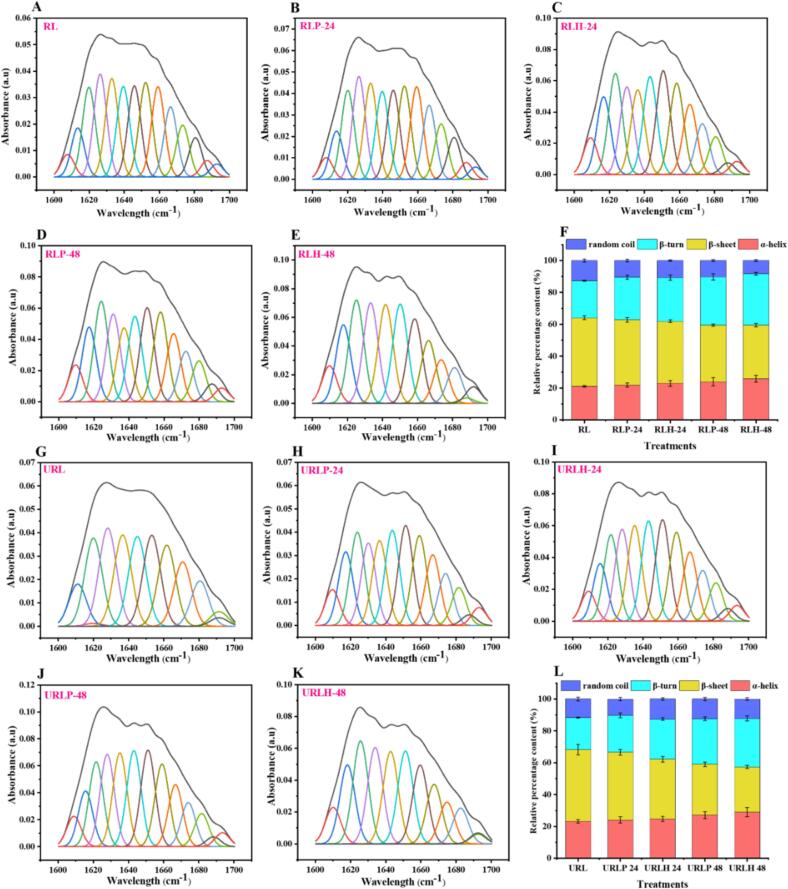
Decomposition (A–E) and relative percentage content (F) of non-sonicated fermented rice lees (RL), and decomposition (G–K) and relative percentage content (L) of TFUT-assisted fermented RL.

The secondary structure of non-sonicated fermented and TFUT-assisted fermented RL was characterized and analyzed using OMNIC and peakfitTM Software after data transformation and deconvolution of amid-I at wavenumbers 1600 to1700 cm^−1^ (Fig. 4). The secondary structures of non-sonicated fermented RL treatments and TFUT-assisted fermented RL treatments were modified after undergoing fermentation and TFUT. The percentage of *α*-helices in RL samples increased from 21.09 to 25.89 % (Fig. 4F) and from 23.19 to 29.03 % (Fig. 4L) for the non-sonicated fermented and TFUT-assisted fermented treatments, respectively. On the other hand, the percentage of *β*-structures decreased from 43.11 to 33.49 % (Fig. 4F) for the non-sonicated samples and from 44.97 to 28.34 % (Fig. 4L) for the TFUT-assisted fermented treatments. Furthermore, when compared to RL, the *β*-turn composition of both non-sonicated fermented and TFUT-treated RL increased. Additionally, upon fermentation, the random coil content of the sample (URLH-24) was found to be higher than other treatments. The *β*-sheet structure is recognized for its relative stability, whereas the *α*-helix, *β*-turn, and random coil structures exhibit greater flexibility [14]. The alterations showed that TFUT-assisted fermentation enlarged and improved the flexibility of RL’s secondary structure. The outcomes observed can be ascribed to the sonication process, which induced the disruption or unraveling of interactions among various regions of protein molecules and local amino acid sequences. The disruption is attributable to various factors, including shear force, shock waves, turbulence, microjets, and free radicals [14].

### 3.6 SEM observation of non-sonicated fermented and TFUT-assisted fermented RL

**Fig. 5:**
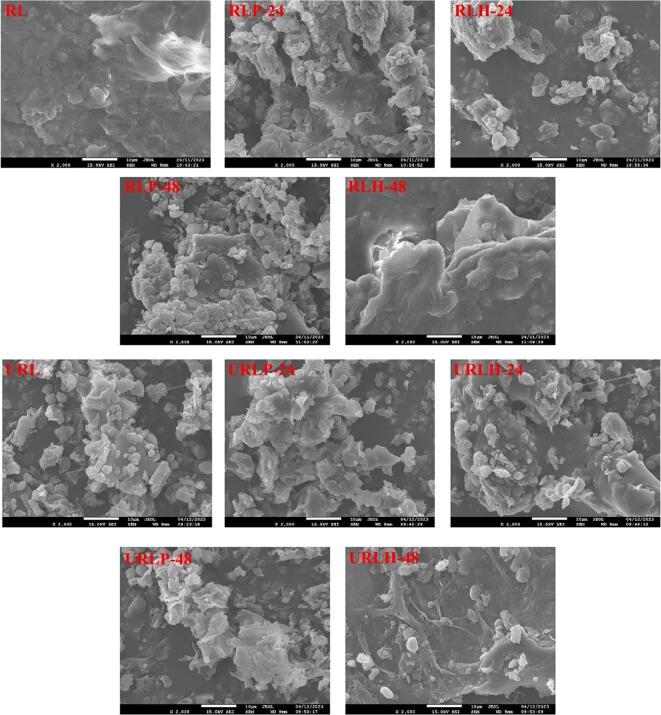
SEM-micrographs of non-sonicated fermented and TFUT-assisted fermented rice lees. * Rows (a, b) and (c, d) represent non-sonicated and TFUT-treated samples, respectively.

SEM images were acquired to examine the effects of fermentation and TFUT on the morphological alterations in the RL, as observed in Fig. 5. Prior to microbial fermentation and TFUT, the RL exhibited an overall irregular and dense structure; afterwards, treatments (fermentation and TFUT) revealed a reduced size and uniform structure, indicating the breakdown of RL particles. Fig. 5 illustrates a more consistent structure and disintegration of components after 48 h of fermentation, in contrast to the samples fermented for 24 h. The URL without fermentation has a more open and consistent structure compared to the control RL. Ultrasounds serve as an efficient mechanism for cellular disruption, attributable to high-speed turbulence, pressure, increased shear stresses, and the generation of primary oxidative radicals, along with various physical phenomena such as cavitation induced by waves [61]. Our findings align with earlier study results [62], which indicated that the structure of Shanlan rice becomes porous and smooth following ultrasound treatment and that the impact of LAB is more pronounced compared to fermented treatments devoid of ultrasound. The ultrasonic produces vapor-filled bubbles in liquid–solid systems; these bubbles swiftly collapse, forming cavities that significantly impact the structure due to the cavitation effect. The disorganized internal structure and surface create an environment that is favorable for bacterial invasion. The collapse of these bubbles triggered violent microjets, which impinged on surfaces and improved porosity by breaking down cellular membranes. A study conducted by [35] showed that ultrasound positively influences the fermentation of hawthorn pulp with LAB, enhancing sugar consumption and acid generation. Even ultrasonic treatment influences the growth of LAB, which in turn leads to an increase in acid production. The cavitation property of the ultrasounds results in the permeability of cell membranes, which increases the movement of nutrients in the matrix, the evacuation of primary and secondary metabolites, and further increases the growth of bacteria. This property of ultrasound is linked with the antioxidant and flavonoid results of RL.

### 3.7 Molecular docking

**Fig. 6:**
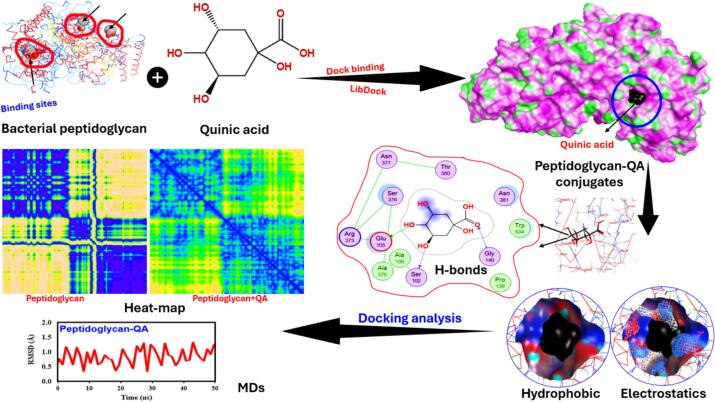
Interaction scheme between peptidoglycan and quinic acid in bacterial cell wall and quinic acid structures, depicting binding affinity, total conjugates, changes in hydrophobicity, electrostatic potentials, H-bonding, heat-map analysis, and MDs-analysis. The binding score and energy affinity of the end conjugates were −1.02 and −1.83 kcal/mol, respectively.

A detailed computational investigation of the interaction between quinic acid (QA) and bacterial peptidoglycan, including molecular docking, molecular dynamics simulation, and structural analysis, is depicted in Fig. 6. The red-circled binding locations on the peptidoglycan molecule in the LibDock docking data indicate that QA interacts with specific spots in the composition of the cell wall. QA establishes hydrogen bonding connections with important residues of amino acids such as Asn, Arg, Glu, and Ser (Fig. 6). The hydrogen bonds suggest that the QA-peptidoglycan conjugate may have a high binding affinity, as they may be essential for maintaining the complex [63]. The complex’s stability may be mostly dependent on the hydrogen bonds, which suggests that the QA-peptidoglycan conjugate may have a high binding affinity. The binding score of −1.02 and an energy affinity of −1.83 kcal/mol suggest a stable interaction, indicating a moderate binding strength that may impact the functional properties of the conjugates. The RMSD graph indicates that the conjugate maintained structural stability throughout the simulation period, further supporting the conclusion that this complex is stable, as corroborated by the subsequent MD simulations. In summary, the analysis of heat maps comparing the native peptidoglycan and its complex with QA, along with MD simulations, indicates that the inhibition of QA binding to the nascent bacterial cell wall will likely require regulation of the essential changes in conformational dynamics observed in this system. Furthermore, the analyses of hydrophobic and electrostatic surface potential offer insights into the regions that play a crucial role in establishing key contacts during the binding process. This indicates that QA may operate ectopically as an effective inhibitor of peptide glycan synthesis or function, thereby supporting a potential mechanism of antibacterial activity. The findings of this study suggest that QA is a compound deserving further exploration in the development of drugs aimed at the bacterial cell wall, as its capacity to disrupt peptidoglycan architecture may inhibit the spread of pathogenic bacteria.

### 3.8 E-nose analysis of non-sonicated fermented and TFUT-assisted fermented RL

**Fig. 7:**
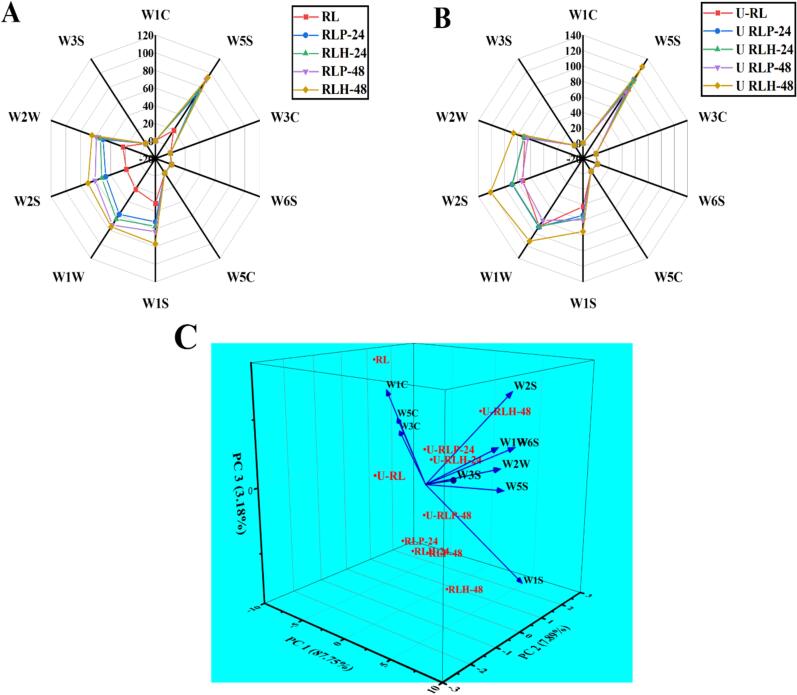
The Radar plot of non-sonicated fermented RL treatments (A), TFUT-assisted fermented RL treatments (B), and PCA analysis (C) among non-sonicated fermented and TFUT-assisted fermented RL treatments.

E-nose analysis is employed to identify the presence and concentration of odor compounds that influence product acceptability among consumers, correlating with sensory evaluation and gas chromatography (GC) results [64]. The flavor profile of RL post-fermentation was characterized through e-nose analysis, highlighting the synergistic effect of TFUT-assisted fermentation. Radar graphs were constructed utilizing the responses from various sensors, resulting in a notable disparity among the treatments. Fig. 7 illustrates that the control samples show elevated readings in the sensors W1C (aromatic organic compounds), W3C (ammonia, which also detects aromatic compounds), and W5C (alkanes, aromatic compounds, and nonpolar organic compounds). RLH-48 exhibits the highest value in sensor W1S, whereas ultrasound-treated fermentation with *L. helveticus* for 48 h demonstrates elevated values across all other sensors (W5S, W6S, W1W, W2W, and W1S), as depicted in Fig. 7A-B. The sensor response for all treatments increased following fermentation and ultrasound treatment, indicating a beneficial effect on the production of flavor compounds. The variation in sensor response values correlates with the differences in the production of volatile flavor compounds, as evidenced by the results of GC analysis [64]. The alterations in RL are associated with intricate biochemical modifications resulting from the application of LAB and ultrasound treatment, which lead to the generation of novel volatile compounds during fermentation [57]. The results align with the findings of [35], which indicated a positive effect of various ultrasound pretreatments on the fermentation performance of LAB using hawthorn pulp. Different volatile organic compounds were produced and increased using ultrasound-assisted fermentation on hawthorn pulp.

Another study demonstrated that the application of triculture LAB in the fermentation of jujube puree resulted in a significant difference in volatile compounds between control and fermented samples [59]. The results of our study show that an increase in fermentation time correlates with higher production and detection of flavoring compounds by the sensors. Treatments fermented for 48 h exhibit a higher concentration of compounds in comparison to both control and 24-h fermented samples. In another investigation, fermentation of brown rice using *Rhizopus oryzae* was conducted for 60 h, revealing an increase in flavoring compounds up to 48 h of fermentation [42]. Various methods exist for presenting and classifying data, with Principal Component Analysis (PCA) being one that relies on the correlation matrix and vector analysis. The rotation of the number axis revealed the disparities between data sets, with the maximum difference associated with dimensional reduction that ultimately generates new axes [65]. Fig. 7C presents the results, indicating that PC1 accounts for 87.75 %, while PC2 and PC3 contribute 7.98 % and 3.18 %, respectively. The results indicate that all fermented and ultrasound treatments are positioned along different axes distinct from the control, demonstrating a significant effect of fermentation using various lactic acid bacteria, duration, and ultrasound treatment on RL.

### 3.9 Sensory evaluation of non-sonicated fermented and TFUT-assisted fermented RL

**Fig. 8:**
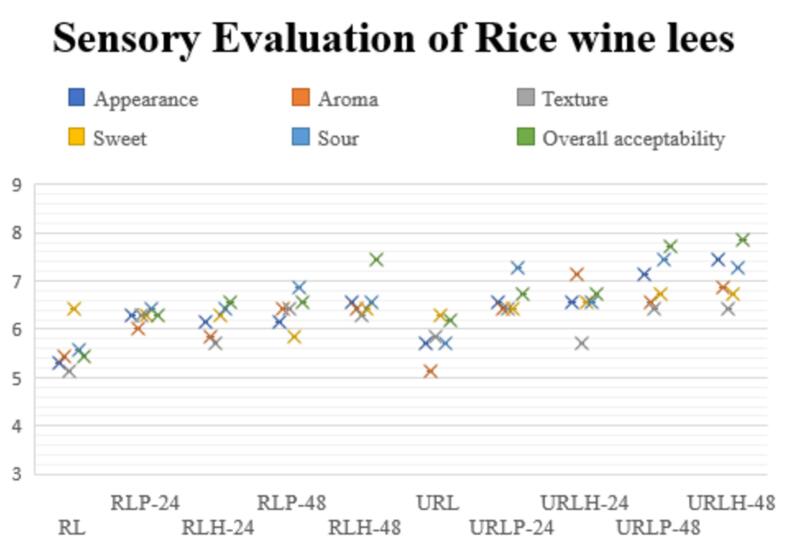
Whisker graph for sensory evaluation of non-sonicated fermented and TFUT-assisted fermented rice lees.

Responses of the product based on sense of smell, hearing, touch, sight, and taste that describe a complex scientific slant is called sensory analysis [66]. In this experiment, sensory analysis was conducted on non-sonicated fermented and TFUT-treated fermented RL, and results were compared with standard (control) RL and presented in the form of a whisker graph (Fig. 8). A nine-point hedonic scale was used, in which 1 represents “dislike extremely” and 9 is attributed to “like very much.“ Fig. 8 shows a significant difference in scores for different parameters like appearance (color), aroma, texture, sweet, sour, and overall acceptability among treatments and in comparison, with the control sample. Appearance mainly represents color of the product; RL is already fermented with yeast during the making process, and during subsequent steps its color changed to yellowish, and during fermentation with LAB and the effect of ultrasound, its color became bright. Fig. 8 represents scores for all the treatments for all parameters that are above five, which shows the acceptability of RL for utilization in any food product; however, application of fermentation and ultrasound increases its acceptability with higher scores and its nutritional value. During fermentation, LAB produces many volatile flavor compounds; scores for the aroma parameter of all the treatments are higher than the raw lees. This phenomenon is associated with the results of E-nose analysis and GC–MS analysis, which show more and more flavoring compounds in 48-h fermented samples. *L. helveticus* has proteolytic activity, and RL is a good source of protein, so more flavor compounds are produced in samples fermented with *L. helveticus*. Noticeable variation was found among all treatments in aroma, flavor (sweet and sour), and overall acceptability. Our findings are supported by [35], who explored the effect of ultrasound-pretreated fermentation on hawthorn pulp by LAB and found a positive impact on sensory parameters of ultrasound-fermented samples and noticed that overall acceptability of the ultrasonicated-fermented hawthorn pulp was more acceptable. In another study, authors formulated the beverage through black tea waste material by fermentation process using *L. plantarum* and concluded that sensory scores for fermented samples increased for 48 and 72 h and then declined at 96 h [67]. URLH-48 achieved the highest level of acceptability, with URLP-48 following closely, suggesting that ultrasound-assisted fermentation for 48 h has a significant effect.

## 4 Conclusion

This work is the first report on the synergistic effects of fermentation and TFUT on the physicochemical, structural, and techno-functional characteristics of both non-sonicated fermented rice lees (RL) and ultrasonicated fermented RL. The techno-functional properties of all treatments were contingent upon the process of TFUT and the time of fermentation. Our findings demonstrate that RL (underutilized valuable material) can be transformed into a value-added product through the integrated processes of fermentation and TFUT. Both TFUT-assisted fermented RL with *L. plantarum* and *L. helveticus* significantly altered the composition of the RL and its secondary structure, as confirmed by FTIR spectra. But TFUT-assisted fermented RL with *L. helveticus* (URLH-48) exhibited enhanced physicochemical properties, leading to an increase in phenolic compounds, a smoother and more uniform texture, and the generation of volatile flavoring compounds that enhance consumer acceptability post-treatment. The techno-functional characteristics of RL were examined, demonstrating that TFUT modified the surface morphology (as evidenced by SEM and molecular docking) and improved the antioxidant activities of the fermented RL samples. The application of TFUT-assisted fermentation with *L. helveticus* improves the nutritional and sensory qualities of rice lees, making them suitable for incorporation in a range of food products. This could expand the range of uses for these overlooked byproducts, which are currently limited to the production of animal feed. The increasing demand for functional ingredients highlights the potential of fermented RL produced through TFUT to be utilized as a functional ingredient in both the food and pharmaceutical industries.

## CRediT authorship contribution statement

**Mian Shamas Murtaza:** Writing – original draft, Methodology, Formal analysis, Conceptualization. **Sanabil Yaqoob:** Writing – original draft, Validation, Formal analysis. **Bismillah Mubeen:** Writing – original draft, Software, Methodology, Investigation. **Aysha Sameen:** Writing – original draft, Validation, Data curation. **Mian Anjum Murtaza:** Writing – original draft, Resources, Investigation, Formal analysis. **Abdur Rehman:** Writing – review & editing, Writing – original draft, Visualization. **Tawfiq Alsulami:** Writing – review & editing, Writing – original draft, Visualization, Validation, Resources. **Sameh A. Korma:** Writing – original draft, Visualization, Validation, Formal analysis. **Ibrahim Khalifa:** Writing – review & editing, Writing – original draft, Validation. **Yong Kun Ma:** Writing – review & editing, Writing – original draft, Supervision, Project administration, Funding acquisition.

## Declaration of competing interest

The authors declare that they have no known competing financial interests or personal relationships that could have appeared to influence the work reported in this paper.
